# Brief Introduction to Cognitive Behavioral Therapy for the Advanced Practitioner in Oncology

**DOI:** 10.6004/jadpro.2017.8.2.6

**Published:** 2017-03-01

**Authors:** Laura Melton

**Affiliations:** University of Colorado Denver School of Medicine, Aurora, Colorado

Advanced practitioners in oncology (APs) are familiar with multidisciplinary and collaborative care; many practice in settings where they refer patients to mental health professionals for the inevitable life distress and potential mental health issues that accompany cancer. Cognitive behavioral therapy (CBT) is commonly used in psycho-oncology, given its empirical base and adaptations for use in the oncology setting. This article succinctly reviews the key structures of CBT and offers practical advice to oncology clinicians on how to use certain techniques in their own practices or as an adjuvant to a mental health referral for their patients. Five specific CBT techniques are reviewed: relaxation, behavioral activation and pleasant event scheduling, thought stopping, positive self-statements, and focus on control.

## WHAT IS CBT?

The phrase "cognitive behavioral therapy," or CBT, refers to a group of interventions that share the notion that psychological distress is sustained by cognitive elements. Pioneered by Ellis (1962) and Beck (1970), the principle of CBT is that maladaptive thoughts promote the continuance of psychological distress and behavioral difficulties. These maladaptive thoughts include the individual’s general beliefs about themself, the world, and the future (Beck, 1970), creating automatic thoughts that may be faulty, inaccurate, or unhelpful in certain situations.

The CBT model crux is that thoughts, behaviors, and emotions are interrelated, and any of those three elements can be a source of distress. Although emotions may be a source of distress, they are the most difficult to change directly. Within the CBT model, maladaptive thoughts and behaviors are challenged to ultimately produce a change in emotional distress.

Utilizing an evidence-based approach, CBT is a time-limited, present-oriented psychotherapy to help people modify dysfunctional thinking and behavior, as a way to help solve current problems (Beck, 1967). Contemporary CBT refers to a family of interventions that blend an assortment of behavioral, emotion-focused, and cognitive techniques (Hofmann, 2011; Hofmann, Asmundson, & Beck, 2013).

## INDICATIONS FOR CBT

In hundreds of clinical trials, CBT has been effective for a wide range of psychological, medical, and psychiatric issues (Butler, Chapman, Forman, & Beck, 2006; Hofmann, Asnaani, Vonk, Sawyer, & Fang, 2012). Based on the standards set forth by Chambless and Ollendick (2001), CBT is considered an empirically supported treatment for a variety of physical and psychological issues, including pain, obesity, schizophrenia, insomnia, anorexia nervosa, binge eating disorder, bulimia nervosa, generalized anxiety disorder, social phobia, chronic headaches, panic disorder, posttraumatic stress disorder, bipolar disorder, depression, and obsessive compulsive disorder. Burgeoning research suggests CBT is helpful in other areas as well, including oncology (Antoni et al., 2006; Chambless & Ollendick, 2001; Compas, Haaga, Keefe, Leitenberg, & Williams, 1998; Fors et al., 2011; Gielissen, Verhagen, & Bleijenberg, 2007; Gielissen, Verhagen, Witjes, & Bleijenberg, 2006; Osborn, Demoncada, & Feuerstein, 2006).

**Focus on Use in Oncology**

The prevalence of distress in patients with cancer is between 30% and 43%, dependent on the type of cancer (Zabora, Brintzenhofeszoc, Curbow, Hooker, & Piantadosi, 2001). Advanced practitioners are recognized as a pertinent part of the cancer care team model and provide significant contributions to patient care (American Society of Clinical Oncology, 2016) by ensuring safe, quality, cost-effective care to patients with cancer (Loftus & Weston, 2001; Vogel, 2003; Volker & Limerick, 2007). To continue to advance the field, APs need to have knowledge and skills to address psychosocial issues that develop in patients with cancer. Examples of APs leading this initiative are numerous (e.g., Daniels, 2015; Hughes, 2016; Petty & Lester, 2014; Yamamoto, 2010).

Cognitive behavioral therapy is a respected form of psychotherapy for which therapists are well trained. It involves having a good skill set, getting to know the patient, and having a much deeper appreciation and understanding of CBT than the brief overview provided in this article. Patients who are depressed or have notable mental health needs should be referred to treatment with a skilled mental health professional. Reading this article is not sufficient to claim expertise in this area, yet attaining an understanding of some CBT techniques can be helpful for non–mental health providers who refer patients to professional psycho-oncology services. Oncology advanced providers who are interested in increasing their education and efficacy in implementing CBT in their clinical work should be encouraged by the literature on non–mental health professionals successfully utilizing CBT with patients when given some focused training (Høifødt, Strøm, Kolstrup, Eisemann, & Waterloo, 2011; Lee, Lim, Yoo & Kim, 2011).

## CBT TECHNIQUES

As CBT and specific techniques are beneficial for patients with cancer (Antoni et al., 2006; Chambless & Ollendick, 2001; Compas et al., 1998; Fors et al., 2011; Gielissen et al., 2006, 2007; Osborn et al., 2006), this article focuses on five techniques that APs can practice in their own lives as well as with patients in conjunction with a referral to a mental health professional: relaxation, behavioral activation and pleasant event scheduling, thought stopping, positive self-statements, and focus on control. Before APs implement these techniques with patients, it is important to make sure this work falls within their specific scope of practice based on their degrees, qualifications, training, state and federal laws, and licensures (Vogel, 2010).

**Relaxation**

Relaxation can help reduce physiological arousal in the body by decreasing the heart rate, respiration rate, blood pressure, muscle tension, metabolic rate, and oxygen consumption (Sapolsky, 1998) and has been found to be helpful for distress in cancer populations (Compas et al., 1998; Tatrow & Montgomery, 2006). When patients with cancer feel relaxed and at ease, they feel more in control. Relaxation also helps us think more rationally, as opposed to emotionally, when we are stressed.

Many specific relaxation techniques exist, including diaphragmatic breathing, guided imagery, various breathing patterns, progressive (and passive progressive) muscle relaxation, meditation, and body scan. Physical issues within patients with cancer may help influence which type of relaxation is best for them; those with breathing difficulties may benefit from non-breathing–focused interventions, so as not to increase their anxiety. Additionally, some people with body aches and pains may benefit more from passive progressive muscle relaxation, as opposed to the traditional progressive muscle relaxation. A quick online search will provide articles, videos, and apps aimed at teaching relaxation. If patients are already comfortable with a specific method, encourage them to increase the frequency of their practice.

Preexisting physical difficulties (e.g., breathing concerns, muscle tension) may be contraindications for using specific relaxation techniques. Consult the patient’s medical history and ask the patient for feedback while doing this exercise together to determine whether or not the relaxation technique chosen is appropriate. Ask patients to practice the agreed-upon relaxation technique for 10 minutes a day, 3 to 5 days per week. Help them problem-solve possible hurdles to practicing. Let them know you will ask them about their progress at the next appointment.

**Behavioral Activation and Pleasant Event Scheduling**

Scheduling and engaging in pleasant events is an effective technique for distress for many people (Cuijpers, van Straten, & Warmerdam, 2007; Ekers, Richards, & Gilbody, 2008; Gawrysiak, Nicholas, & Hopko, 2009; Mazzucchelli, Kane, & Rees, 2009; Sturmey, 2009), including those with a cancer diagnosis (Hopko et al., 2011). When people are feeling down, they can lose motivation and interest in things they usually enjoy doing; increasing activity and engaging in pleasant events are important to increase positive emotions.

It is important to encourage people to think of a variety of activities, as it can be difficult for patients to come up with a list on their own when their life or outlook has changed. Physical and cognitive changes may make it difficult to engage in activities that patients enjoyed before a cancer diagnosis.

Encourage patients to contemplate big and small events, as well as those with a wide range of cost. Finally, urge commitment to engaging in an event and scheduling it within the next week and follow up on their progress when you see them next in the clinic.

A history of depression or acknowledgment of current symptoms of depression may indicate a contraindication to trying this technique and the potential need for referral to a mental health professional. It is important to remind patients that one particular technique is not a panacea for their cancer distress. This technique can lead to downplaying the current psychological struggles patients are experiencing; many patients with cancer are already bombarded with family and friends telling them to "think positive," and therefore they may not benefit from this technique if they are not appropriately instructed.

**Thought Stopping**

Originally introduced in 1928 (Bain, 1928), thought stopping gained popularity in the 1950s, based on the work of Joseph Wolpe and other behavior therapists interested in decreasing, and ultimately eliminating, unhelpful thoughts or behaviors (Cautela & Wisocki, 1977; Davis, Eshelman, & McKay, 2008; Zinsser, Bunker, & Williams, 2010). The process includes first identifying a negative, anxious, or unhelpful thought. Each time a client notices having the target thought, they say "stop" or intervene in some way. Alternative interventions include keeping a tally of the behavior and gently snapping a rubber band on their wrist when they have the thought.

As these thoughts often happen automatically and without much intention, it takes time to change the thought process. Initially, people might not notice the target thought until after it has passed. With thought stopping, individuals gradually become aware of having the target thought sooner, until they are able to identify the point before they have the thought. Thought stopping can be helpful for patients with cancer who may sometimes think that their behavior caused their cancer or that they are somehow to blame because they have cancer.

Thought stopping is not effective in all cases. It is recommended more often when the problem is primarily cognitive (a thought) and when the thought is considered painful or directing the individual to an uncomfortable emotional state (Davis et al., 2008). Contraindications include a lack of buy-in (which is not uncommon) from the patient, as thought stopping can only work if it is used continuously and consistently for a period of time.

Ask patients to commit to practicing, and explore possible obstacles to their success. Many people benefit by having reminders of their desire to change their thoughts in key locations (such as sticky notes). Assess progress through patient-reported frequency of the target thought; ask patients to keep a small tally with them to mark down the frequency of the behaviors each day to quantify their progress.

**Positive Self-Statements**

Another technique to fight negative thoughts is to intentionally think good thoughts (Hardy, 2006; Moran, 2012; Oliver, Markland, & Hardy, 2010; Todd, Hardy, & Oliver, 2011). Positive self-statements promote encouragement and motivation (Prasertsri, Holden, Keefe, & Wilkie, 2011). They should be short and believable to the individual.

Similar to behavioral activation and pleasant event scheduling, the positive self-statement technique can be a target for critics of the "think positive" movement. It is important to instruct patients that this is not a Pollyanna approach. It does not downplay their current circumstances or the negative things in their life. Patients are given permission to intentionally think a thought that makes them feel good or encouraged. Research suggests that using positive self-statements works best for individuals who already have high self-esteem, as it can make people with low self-esteem feel worse (Wood, Perunovic, & Lee, 2009); therefore, low self-esteem is a contraindication for using this technique.

If you choose to implement this technique, be sure to help patients generate their positive self-statements, but make sure they find the statement to be believable and helpful. If someone has difficulty generating a statement, prompt them with poignant starter questions:

- What are you most proud of about yourself?
- What are your strengths, which you may forget in stressful situations?
- When were you proud of the way you handled a stressful situation?
- Why do people praise or compliment you?

Ask patients to write their positive statements on an index card they carry with them, and suggest they practice using the statement daily. At the next clinic visit, check on how this technique is working for them.

**Focus on Control**

Every situation has aspects that are controllable and aspects that are uncontrollable. When a person is overwhelmed or helpless, it is especially important to identify what aspects can be controlled. Perceived control sharply declines after a cancer diagnosis, regardless of the prognosis (Ranchor et al., 2010). Following a cancer diagnosis, those individuals who are not able to regain somewhat of their general sense of control over life do worse emotionally (Ranchor et al., 2010).

Help patients differentiate between controllable (e.g., whether to do a medical procedure) and uncontrollable (e.g., a new cancer diagnosis) aspects. Focus on things that can be controlled, and change an aspect of the situation: It might be seeking information, making a decision, or communicating with others. Help them to think about behaviors and thoughts that they have control over; perhaps they want to make some changes. For the areas in which they have no control, help them to focus on their reactions instead. Patients may be able to practice relaxation, use positive self-statements, or distract themselves with something pleasant.

For patients who state "I have no control over my cancer," start with praising them for taking control of their health by coming to the appointment. Point out that they chose to come and seek medical care, whereas other people may choose not to do so. Patients who are in denial about their cancer or who use the setup of this technique as an opportunity to engage in an argument about their inability to control anything should be steered clear of this technique. The goal is not to force someone to radically change his or her thinking, but to open the possibility that there is some small thing that can be controlled.

## SUGGESTIONS FOR APS

Identifying the reason for the visit will be the first step in determining which, if any, of these CBT techniques would be appropriate for a particular patient. Start small by suggesting one technique based on the indications mentioned in this article. Practice the technique together in session, answer questions about the technique so the patient feels comfortable, and ask the patient to practice the technique as homework. Plan to check on the success of the homework at the next follow-up session.

Patients who may meet the criteria for a mental health diagnosis require a psychological assessment prior to an unlicensed mental health provider trying any techniques. Similarly, it is better not to attempt these techniques than to do so when you do not have the proper time, skills, or knowledge to properly teach them to a patient.

## CONCLUSION

Cognitive behavioral therapy is helpful for a variety of physical and psychological issues, including coping with the emotional stress of cancer. Mental health professionals trained in CBT are appropriate referral sources for patients who have cancer, but as APs, there are aspects of CBT that can be helpful to practice with patients in adjunct to a mental health referral. Relaxation, behavioral activation and pleasant events, thought stopping, positive self-statements, and focus on control are all broad, easy-to-implement techniques.
